# High levels of genetic variability and differentiation in hilsa shad, *Tenualosa ilisha* (Clupeidae, Clupeiformes) populations revealed by PCR-RFLP analysis of the mitochondrial DNA D-loop region

**DOI:** 10.1590/S1415-47572009005000023

**Published:** 2009-01-30

**Authors:** Sabuj Kanti Mazumder, Md. Samsul Alam

**Affiliations:** Department of Fisheries Biology and Genetics, Bangladesh Agricultural University, MymensinghBangladesh

**Keywords:** genetic variability, PCR-RFLP, mtDNA, *Tenualosa ilisha*

## Abstract

The hilsa shad, *Tenualosa ilisha* (Clupeidae, Clupeiformes) is an important anadromous clupeid species from the Western division of the Indo-Pacific region. It constitutes the largest single fishable species in Bangladesh. Information on genetic variability and population structure is very important for both management and conservation purposes. Past reports on the population structure of *T. ilisha* involving morphometric, allozyme and RAPD analyses are contradictory. We examined genetic variability and divergence in two riverine (the Jamuna and the Meghna), two estuarine (Kuakata and Sundarbans) and one marine (Cox's Bazar) populations of *T. ilisha* by applying PCR-RFLP analysis of the mtDNA D-loop region. The amplified PCR products were restricted with four restriction enzymes namely, *Xba*I, *Eco*RI, *Eco*RV, and *Hae*III. High levels of haplotype and gene diversity within and significant differentiations among, populations of *T. ilisha* were observed in this study. Significant F_ST_ values indicated differentiation among the river, estuary and marine populations. The UPGMA dendrogram based on genetic distance resulted in two major clusters, although, these were subsequently divided into three, corresponding to the riverine, estuarine and marine populations. The study underlines the usefulness of RFLP of mtDNA D-loop region as molecular markers, and detected at least two differentiated populations of *T. ilisha* in Bangladesh waters.

## Introduction

The hilsa shad, *Tenualosa ilisha,* belonging to the sub-family Alosinae of the family Clupeidae (Clupeiformes, Pisces), occurs in foreshore areas, estuaries, brackish-water lakes and freshwater rivers of the western division of the Indo-Pacific faunistic region. Its marine distribution extends from Iran and Iraq in the Persian Gulf to the west coast of India in the Arabian Sea and the Bay of Bengal (Pillay and Rosa Jr, 1963).

Hilsa shad is the largest single fishable species in Bangladesh, present in almost all the major river systems, estuaries and marine environments (Bay of Bengal), and at present contributing to approximately 12% of the total fish production and 20% of fishery-capture (inland and marine) with a biomass of 78, 273 metric tons from inland fisheries and 198, 850 metric tons from marine (DoF, 2006). Hilsa is termed the national fish of Bangladesh, due to its popularity and economic importance. However, the production of hilsa in Bangladesh has waned when compared to earlier estimates. Until 1972, hilsa shad fishery was prosperous upstream in the rivers of Bangladesh, especially in the Padma and Meghna. Fishery has entered into a severe decline upstream and is nowadays mainly concentrated downstream, as well as in estuaries, coastal areas and the sea (Nurul Amin *et al.*, 2004). Due to the low water discharge from the upstream Ganges at the Farakka barrage (in West Bengal, India) with the consequent heavy siltation, the indiscriminate exploitation of juveniles (*Jatka*), disruption of migration routes, loss of spawning, feeding and nursing grounds, increased fishing pressure, etc., have all contributed to this decline.

The hilsa shad is largely an anadromous species, but two other ecotypes - a fluvial potamodromous type and a marine type - have been recognised. The potamodromous stocks appear to remain in the middle reaches of the rivers throughout the year and breed therein. The anadromous stocks, whose normal habitat is the lower region of the estuaries and the foreshore areas, ascend the rivers during the breeding season and return to the original habitat after spawning (Raja, 1985). The upstream migration during the main breeding season depends largely on the commencement of the south-west monsoon and consequent flooding of the major rivers of Bangladesh, Burma and India. However, the exact spawning season for the species is still controversial, as spawning varies from a few months to all the year round. However, it is not known whether migratory populations mix during migration or whether they pass each other spatially and temporally. Therefore, the exact stocks are still in dispute.

Reports on the stock structure of the valuable tropical shad *T. ilisha*, based on samples collected from Bangladesh and India and through morphometric, allozyme and RAPD analyses, are contradictory. Dahle *et al.*(1997) claimed to have discriminated three populations of hilsa shad in Bangladesh waters, such as Chandpur (Meghna river), Barguna (Brackish water, estuarine) and Cox's Bazar (sea water) by using RAPD markers. On the basis of a single polymorphic locus, *PGM*, Rahman and Naevdal (2000) claimed that there existed two separate gene pools of hilsa shad in Bangladesh waters. Milton and Chenery (2001), on the basis of otolith chemistry and morphometry, and Salini *et al.* (2004), on the basis of allozyme and morphometric analyses, inferred, however, that there was a single stock of hilsa in Bangladesh waters, this including the Bay of Bengal. Brahmane *et al.* (2006) identified two groups of *T. ilisha* in India, one comprising the Ganges and Yamuna rivers and the other the Hooghly and Narmada rivers, by using RAPD markers.

Among the different markers available for population genetic analysis, differences in mitochondrial DNA are probably the most widely used, since they follow maternal inheritance, do not undergo rearrangements or recombination and present higher mutation rates than those of nuclear genes (Avise, 2004). RFLP analysis of the mtDNA control region is a simple technique for revealing genetic variation in the mitochondrial genome of an organism.

In this study, we analyzed haplotype and gene diversity in five samples of *T. ilisha*, using PCR-RFLPs of the mtDNA D-loop region as genetic markers. The aim of the present study was to compare mtDNA genetic diversity in *T. ilisha* samples collected from rivers, estuaries and the sea, and to delineate genetic differentiation among populations of the three major aquatic environments. We also report here whether there are any genetic differences between distant river populations of *T. ilisha*, as well as between estuarine populations. The purpose of the present study is to examine the usefulness of mitochondrial D-loop region diversity to supplement allozyme and RAPD data on the population genetic structure of *T. ilisha*.

## Materials and Methods

### Fish samples and extraction of DNA

**Figure 1:**
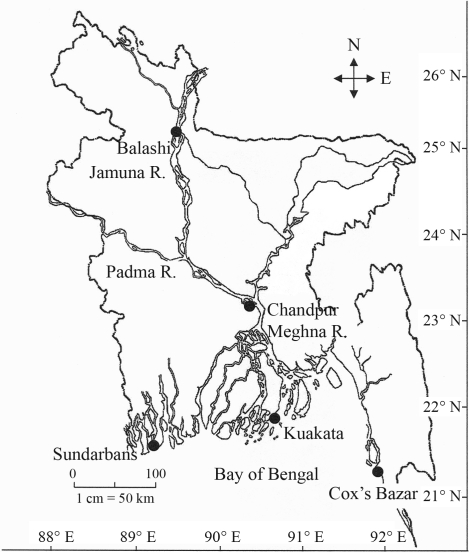
Map of Bangladesh showing the five sites (•) from where the samples of *T. ilisha* were collected. The three big rivers, the Padma, Jamuna and Meghna, are also shown. These three rivers constitute the major riverine fishery of *T. ilisha* in Bangladesh. R: river.

Ninety hilsa (*T. ilisha*), with an average body weight of about 400 g, were collected from five geographic locales: Balashi (Gaibandha, upstream of the Jamuna River)), Chandpur (middle of the Meghna River), Kuakata (estuaries), Sundarbans (estuaries) and Cox's Bazar (Bay of Bengal) with the help of artisanal fishers (Figure 1). Immediately after landing the fish on board, a piece of dorsal fin from each was carefully collected and preserved in 95% ethanol. The samples were stored at -20 °C until further use for the extraction of DNA. Total DNA (nuclear + mitochondrial DNA) of each individual fish was extracted from approximately 50 mg of fin tissue by standard proteinase-K digestion, phenol:chloroform:isoamyl alcohol extraction and the alcohol precipitation method as described by Islam and Alam (2004). The quality of the DNA samples was checked by electrophoresis on 1% agarose gel and the quantity determined by using a spectrophotometer, prior to PCR amplification of the mtDNA D-loop region.

### Amplification of mitochondrial DNA

The 2.2 kb D-loop region of the mtDNA, including a part of the cytochrome b and 12SrRNA genes, was amplified from each of the 90 samples by using the primer-pairs L-12260 (5' → 3', CAT ATT AAA CCC GAA TGA TAT TT) and H-1067 (5' → 3', ATA ATA GGG TAT CTA ATC CTA GTT T) (Martin *et al.*, 1992). The mtDNA fragment was amplified in a reaction volume of 30 μL containing 75 ng template DNA, 3 μL of 10X buffer (100 mM Tris-HCl, pH = 8.3, 15 mM MgCl_2_, 500 mM KCl), 3 μL of 2.5 mM dNTPs (dATP, dCTP, dGTP, dTTP), 1 μM of each primer and 2 units of Taq DNA polymerase (GENEI Bangalore, India). An oil-free thermal cycler (Master Cycler Gradient Eppendorf, Germany) was used for PCR amplification with the following cycle parameters: initial denaturation at 94 °C for 3.0 min, followed by 35 cycles of denaturation at 94 °C for 45 s, annealing at 45 °C for 45 s and extension at 72 °C for 2.0 min. A final extension step at 72 °C for 7 min followed the last cycle. The PCR products were confirmed by electrophoresis on agarose gel and subjected to digestion with restriction endonucleases.

### RFLP analysis

Four restriction enzymes (*Xba*I*, Eco*RI*, Eco*RV and *Hae*III) were used for digesting the amplified fragments. Ten microlitres of the PCR product were used for each digestion following manufacturer's (Promega) recommended conditions. The restricted PCR products were electrophoresed on 1.4% agarose gel containing ethidium bromide and photographed under UV light using a Geldoc camera (UVP Inc). Two molecular weight markers, λ-DNA-*Hind*III digest and 100 bp DNA ladder were run on each gel along with the digested PCR products. The sizes of mtDNA restriction fragments were measured by using the software, DNAfrag (Nash, 1991).

### Genetic data analysis and dendrogram construction

Restriction patterns generated from each restriction endonuclease were given letter designations in the order of their frequencies. Haplotype A refers to the most common digestion pattern in the analyzed samples. The remaining alphabetical profile names (B, C, etc.) indicate variant digestion patterns reflecting their frequencies in order. Composite haplotypes were constructed from all the enzymes used and arranged from the enzyme generating the fewest polymorphic patterns to that generating the most (*Xba*I, *Eco*RI, *Eco*RV and *Hae*III in this order). The presence or absence of restriction sites in the control region was inferred for each of the four enzymes from a series of restriction fragment patterns. The site codes across the amplified region for a restriction enzyme were concatenated and each fish was assigned a code of four letters that described its composite, multi-enzyme haplotype.

A single data matrix comprising the composite haplotypes and their frequencies in the five populations was constructed. Haplotype and gene diversity, both calculated as per Nei (1987), and a hierarchical analysis of population subdivision performed using the analysis of molecular variance with 1000 simulated samples (AMOVA, Excoffier *et al.* 1992), were implemented in ARLEQUIN v. 3.1 (Excoffier *et al.*, 2005). Both the Tajima (1989) D-test and the Fu (1997) Fs-test were applied to test deviations from neutral molecular evolution. Significance was assessed in both cases by generating random samples (number of simulated samples: 1000) under the hypothesis of selective neutrality and population equilibrium, as implemented in ARLEQUIN. Significance levels of pair-wise F_ST_, under the hypothesis of no differentiation between populations, were determined by means of 10000 permutations of haplotypes between populations. Genetic distance values between population-pairs were calculated from the F_ST_ values of the respective population-pair by using the formula: D (genetic distance) = -log(1 - F_ST_) (Reynolds *et al.*, 1983). Distance data were used to draw up an unweighted pair-group method of averages (UPGMA) dendrogram by using MEGA version 4 (Tamura *et al.*, 2007) software.

## Results

PCR amplification of the D-loop region resulted in a product of approximately 2.2 kb with no detectable size differences between *T. ilisha* samples. Using this sequence, four restriction enzymes (*Xba*I, *Eco*RI, *Eco*RV and *Hae*III) were selected to obtain RFLP markers. All enzymes produced polymorphic banding patterns and restriction sites. Restriction of the PCR fragment with the four restriction endonucleases resulted in a total of 35 restriction profiles in the samples collected from five locations in Bangladesh. The cleavage patterns and estimated lengths of the restriction fragments for different restriction enzymes are shown in Table 1. The cleavage patterns produced due to variations in restriction sites were three for *Xba*I and *Eco*RI, five for *Eco*RV and seven for *Hae*III. In some cases, however, the sum of the fragment sizes did not exactly equal the total size of the amplified region, probably due to small fragments being lost or bands of similar size co-migrating. The 35 different haplotypes (composite genotypes) detected in 90 individuals of the five populations, along with their numerical frequencies, are presented in Table 2.

Out of the 35 haplotypes, only two, VI and XI, were distributed randomly among the five populations, the others being either specific for a particular population or shared by two or three (mostly two) populations (Table 2). Haplotypes II, III, IV, V, VII, IX and X were specific to the Balashi population, XII and XIII to the Chandpur, XVII, XVIII, XIX, XXI, XXIII, XXIV, XXV and XXVI to the Cox's Bazar, XXVII, XXVIII, and XXIX to the Sundarbans and XXXV to the Kuakata. Haplotype I was shared by the Balashi, Chandpur and Sundarbans populations, VIII by the Balashi and Chandpur, XVI by the Chandpur and Sundarbans and XX by the Cox's Bazar and Kuakata. Haplotype I was dominant in the Balashi and Chandpur population, haplotype VI in the Chandpur, haplotypes XVII and XX in the Cox's Bazar and XXVIII in the Sundarbans. None of the haplotypes were found to be dominant in the Kuakata population.

Polymorphic haplotypes were observed in all the five populations. The rates of haplotypes (number of haplotypes observed divided by the sample size) ranged from 0.500 (Chandpur) to 0.722 (Sundarbans) (Table 3). Haplotype diversity was high in all the five populations, ranging from 0.882 (Chandpur) to 0.960 (Sundarbans). The average gene diversity across the loci was highest in the Sundarbans population (0.569) and lowest in the Kuakata (0.343). The overall Fixation Index (F_ST_) across the populations was 0.092 and the population specific F_ST_ indices ranged from 0.085 (Sundarbans) to 0.105 (Chandpur). Tajima's D test (Tajima, 1989) and Fu's F test (Fu, 1997) were not significant (p > 0.05) in all the populations.

**Figure 2:**
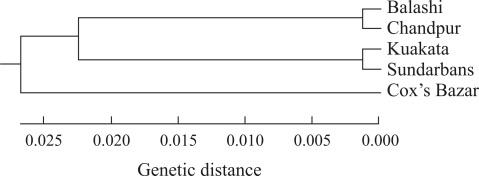
UPGMA dendrogram summarising the genetic distance between pairs from the five different populations of *T. ilisha*.

Variance components and F-statistics analogs (Phi-st) were calculated with AMOVA. No significant differences among samples were found. Hierarchical analyses indicated that 90.78% variation was contained within local populations while 9.22% was distributed among the populations. However, significant differentiation (F_ST_) values were found among the five populations of *T. ilisha*. The F_ST_ value between the Chandpur and Sundarbans populations was the highest and that between the Chandpur and Balashi the lowest. The F_ST_ values between the Balashi-Chandpur, Cox's Bazar-Kuakata, and Sundarbans-Kuakata population pairs were insignificant, while these values between the remaining seven were found to be significant (Table 4). Genetic distance values calculated from the corresponding F_ST_ values between the population-pairs are shown in Table 4. The highest genetic distance was observed between the Chandpur and Sundarbans population, while the lowest was observed between the Chandpur and Balashi population. The UPGMA dendrogram based on genetic distance resulted in two clusters. The first cluster contained four populations and the second contained only the Cox's Bazar population. The first cluster was subsequently divided into two sub-groups. The two river populations, Balashi and Chandpur, formed one group and the two estuarine populations, Kuakata and Sundarbans, the second (Figure 2).

## Discussion

Mitochondrial DNA-RFLP has been proved to be an effective technique for population discrimination. The present study was an attempt to reveal genetic variability and stock discrimination of hilsa populations in Bangladesh waters, including freshwater, estuarine and marine environments, by PCR mtDNA RFLP analysis.

The detection of 35 different haplotypes in only 90 individuals of five *T. ilisha* samples underlines the usefulness of RFLPs of the D-loop region as molecular markers for investigating the geographic structure of the species. High diversity indices were obtained within each sample (Table 3). The average haplotypic diversity in *T. ilisha* observed in the present study, fell within the upper part of the range (0.473-0.998) for some other fishes as reported by Avise *et al.* (1989), and reached as high as that (0.892) obtained in red seabream collected from four locations of western Japan through PCR-RFLP analysis of the mtDNA D-loop region by Tabata and Mizuta (1997). However, since the estimation of haplotype diversity is based on haplotype frequencies alone, it is dependent on the number of restriction enzymes used (Graves and McDowell, 1994). These results suggest that genetic variability in *T. ilisha* is quite high. High levels of genetic diversity appear to be commonly observed in migratory fishes with large panmictic populations (Santos *et al.*, 2007). This is because large effective population size and high migration rates minimize the effect of genetic drift as a force that lowers intra-population genetic variability. However, the presence of private alleles indicates that these populations are not completely panmictic. The non-significant values (p > 0.05) obtained from Tajima's D and Fu's Fs statistical tests indicate that the sampled populations of *T. ilisha* are in genetic equilibrium, which means that apparently there is no pressure of selection on the population with regard to mitochondrial DNA haplotypes.

The test for population differentiation gave significant p-values (p < 0.05), indicating that composite haplotypes were not distributed randomly with respect to locality. F-statistics (Weir and Cockerham, 1984) yielded an overall F_ST_ value of 0.092 which indicates that genetic exchange occurring among the populations was not sufficient to prevent either genetic differentiation or structuring into genetically differentiated subpopulations in *T. ilisha* in Bangladesh. Geographic differentiation of the hilsa shad *T. ilisha* has also been reported previously. Brahmane *et al.* (2006) identified two groups of hilsa in India, one comprising the Ganges and Yamuna rivers and the other the Hooghly and Narmada rivers. Dahle *et al.* (1997) reported the existence of three stocks of hilsa shad in Bangladesh waters, namely the Chandpur (Meghna river), Barguna (Brackish water, near Kuakata)) and Cox's Bazar (salt water), when using RAPD markers. Our results suggest there are at least two, one marine, and the other riverine-estuarine, or possibly three (riverine, estuarine and marine), stocks of hilsa in Bangladesh. The second possibility complies with the hypothesis of three groups of hilsa shad, anadromous, potamodromous and marine, as reported by Pillay and Rosa Jr (1963). Now, the question is whether the *T. ilisha* feeding in the Chandpur area migrate further upstream to spawn in the Padma and the Jamuna rivers or not. No significant difference in F_ST_ value between the samples from Balashi (the Jamuna) and Chandpur (the Meghna) suggest a single panmictic riverine population of *T. ilisha* in Bangladesh. However, this conclusion may be contradicted through the presence of some private haplotypes in the Balashi and Chandpur populations. These results also run counter to those of Shifat *et al.* (2003), who reported that the two river populations of *T. ilisha*, the Meghna and the Padma were different stocks, though they observed certain alleles shared by some individuals of these two river samples. The presence of private haplotypes indicates the extent of mixing between populations. Due to their locations (Figure 1) and the mode of migration of the species, the possibility of mixing in the Chandpur (the mid Meghna river), Kuakata and Sundarbans populations is higher, reflected by the lower number of private haplotypes in these populations. Higher numbers of private haplotypes have been obtained in the two most distant populations, Balashi and the Cox's Bazar (Table 2), which indicates that the low number of samples is not the main cause of the high number of private haplotypes in these populations. Therefore, we would like to opine that certain differences exist among the river populations, perhaps due to preferential movements of a fraction of the migratory groups, although the difference is not high enough to distinguish the populations as separate stocks (sub-populations).

We observed significant differentiation between the riverine and marine (Cox's Bazar) populations, but not between the marine and one of the estuarine. There is no evidence of spawning in the sea, but there is evidence of *T. ilisha* estuarine spawning in Bangladesh (BOBP, 1987). Thus, fishes from the Cox's Bazar region must migrate to any of the rivers or at least up to the estuaries. Our results suggest that fishes from the Cox's Bazar region may go up to the Kuakata region, but not to the Meghna and Jamuna rivers, as significant differentiations between the Cox's Bazar and the two river populations have been observed, but not between the Cox's Bazar and Kuakata (Table 4).

The five samples were grouped into two clusters on the basis of genetic distance among population-pairs. Nevertheless, the two clusters were finally divided into three groups corresponding to the three different environments, riverine, estuarine and marine (sea). The slight distance between the Balashi and Chandpur population indicates their belonging to the same stock. However, the sharing of two haplotypes by all the populations has led us to postulate the presence of a certain degree of gene flow among those studied.

Hilsa shad (*T. ilisha*) is the national fish of Bangladesh. The delicious taste and contribution of this fish has lead to its position as one of the most economically important fish in this country. Furthermore, hilsa fishery constitutes the largest single species fishery in the riverine, estuarine and marine ecosystems of the country. It is essential to recognize that geographically and genetically different populations as different stocks should be managed separately (Carvalho and Hauser, 1994). Our studies on mtDNA PCR-RFLP in *T. ilisha* indicate that population sub-division does indeed exist in this species. On the basis of the AMOVA, we can conclude that there are three stocks of hilsa with a substantial level of inter-population genetic divergence among river, estuarine and marine populations. The high level of haplotype variability found in only five populations underlines the usefulness of RFLPs of the D-loop region as molecular markers to investigate the geographic structure of *T. ilisha*. We analyzed only 18 individuals from each population, and the electrophoretic analysis of restriction fragments is also not so perfect as sequencing the PCR product. Therefore, to reach a more definite conclusion, larger samples from throughout the distribution range of the species in the country should be analyzed by sequencing the mtDNA D-loop region and/or with faster evolving molecular markers, such as micro-satellite loci.

## Figures and Tables

**Table 1:** Restriction patterns for different haplotypes in the 2.2 kb mtDNA D-loop fragment from ninety *T. ilisha* collected from five locations in Bangladesh (values show restriction fragment in bp).

		Haplotypes						
Restriction enzyme	Recognition sequence	A	B	C	D	E	F	G
*Xba*I	T/CTAGA	1608	1241	2168				
		568	930					

*Eco*RI	G/AATTC	1439	1566	1529				
		566	566	566				
		95						

*Eco*RV	GAT/ATC	1432	1296	1296	1375	1432		
		707	707	675	707	675		

*Hae*III	GG/CC	680	680	680	680	1210	680	849
		660	660	660	660	580	660	680
		440	541	409	580	200	409	660
		425	200	390	200		200	

**Table 2:** Geographic distribution of 35 composite mtDNA D-loop region haplotypes among five conspecific populations of *T. ilisha* based on four restriction enzymes (*Xba*I, *Eco*RI*, Eco*RV, and *Hae*III) (relative frequencies are in parentheses).

Composite haplotypes			Geographic distribution of mtDNA composite haplotypes				
			Balashi	Chandpur	Cox's Bazar	Sundarbans	Kuakata
I	AAAA		3(0.167)	4(0.222)	0	1(0.056)	0
II	AAAB		1(0.055)	0	0	0	0
III	ABAC		1(0.055)	0	0	0	0
IV	AAAC		2(0.111)	0	0	0	0
V	ABCC		2(0.111)	0	0	0	0
VI	AABB		2(0.111)	5(0.278)	1(0.055)	1(0.055)	2(0.111)
VII	ABBB		1(0.055)	0	0	0	0
VIII	AAAE		1(0.055)	1(0.055)	0	0	0
IX	AACA		1(0.055)	0	0	0	0
X	AABB		2(0.111)	0	0	0	0
XI	AADA		2(0.111)	1(0.055)	1(0.055)	1(0.055)	1(.055)
XII	ABAD		0	1(0.055)	0	0	0
XIII	ACAA		0	1(0.055)	0	0	0
XIV	AABC		0	2(0.111)	0	0	2(0.111)
XV	AADB		0	1(0.055)	0	0	1(0.055)
XVI	AABE		0	2(0.111)	0	1(0.055)	0
XVII	AACB		0	0	3(0.167)	0	0
XVIII	AACF		0	0	2(0.111)	0	0
XIX	AACC		0	0	1(0.055)	0	0
XX	CBBA		0	0	4(0.222)	0	2(0.111)
XXI	BADA		0	0	1(0.055)	0	0
XXII	ABCD		0	0	1(0.055)	1(0.055)	0
XXIII	CACA		0	0	1(0.055)	0	0
XXIV	BACA		0	0	1(0.055)	0	0
XXV	AABG		0	0	1(0.055)	0	0
XXVI	AADC		0	0	1(0.055)	0	0
XXVII	BACB		0	0	0	2(0.111)	0
XXVIII	BADE		0	0	0	3(0.167)	0
XXIX	BABA		0	0	0	2(0.111)	0
XXX	BABB		0	0	0	2(0.111)	1(0.055
XXXI	ABAE		0	0	0	1(0.055)	2(0.111)
XXXII	AADG		0	0	0	1(0.055)	2(0.111)
XXXIII	BAAE		0	0	0	1(0.055)	2(0.111)
XXXIV	BADC		0	0	0	1(0.055)	2(0.111)
XXXV	ABDB		0	0	0	0	1(0.055)

**Table 3:** Genetic variability within the five different populations of hilsa shad (*T. ilisha*).

	Populations				
	Balashi	Chandpur	Cox's Bazar	Sundarbans	Kuakata
Sample size	18	18	18	18	18
N. of haplotypes	11	9	12	13	11
N. of polymorphic sites	20	20	23	24	19
Rate of haplotype	0.611	0.500	0.667	0.722	0.611
Haplotype diversity	0.948 ± 0.05	0.882 ± 0.05	0.967 ± 0.03	0.960 ± 0.03	0.954 ± 0.02
Expected heterozygosity (gene diversity)	0.451 ± 0.29	0.363 ± 0.26	0.531 ± 0.16	0.569 ± 0.24	0.343 ± 0.09
Population specific F_ST_ indices	0.099	0.105	0.092	0.085	0.079
Tajima's D test	0.751	0.150	1.280	0.082	0.565
Fu's Fs test	-1.057	0.178	-3-26	-1.899	2.814

**Table 4:** Estimates of pairwise F_ST_ (Population differentiation) values (below diagonal) and genetic distance (above diagonal) among five *T. ilisha* populations in Bangladesh. The F_ST_ values were calculated with 110 permutations.

	Ba	Ch	CB	Su	Ku
Ba	0.00000	0.00243	0.04816	0.07628	0.01959
Ch	0.00563NS	0.00000	0.07252	0.07847	0.03334
CB	0.10497***	0.15380***	0.00000	0.05637	0.03402
Su	0.16109***	0.16531***	0.12173***	0.00000	0.00145
Ku	0.044120*	0.07391*	0.07536NS	0.00334NS	0.00000

Note: Ba: Balashi, Ch: Chandpur, CB: Cox's Bazar, Su: Sundarbans, Ku: Kuakata, NS: Not significant; *: p < 0.05; ***: p < 0.000.
